# Supplementary material for: Novel Mutation in the Feline NPC2 Gene in Cats with Niemann–Pick Disease
Source: Animals (Basel). 2023 May 24;13(11):1744. doi: 10.3390/ani13111744 (PMC10252137; doi:10.3390/ani13111744)
Supplement: Supplementary file 1 [file animals-13-01744-s001.zip › animals-2347701-supplementary.pdf]

Table S1. Primers used to amplify exons and splicing regions of the feline *NPC1* gene.

Exon no.	Name	Primer sequence (5′→3′)	Length (mer)	GC (%)	T_m (ºC)	Amplicon (bp)	T_a (ºC)
1	fNPC1-E1-F	AACCGCACCAAGGCAGCATG	20	60	57.7	133	56
	fNPC1-E1-R	TAGCCTCCCGGCCCTCCTC	19	74	57.9		
2	fNPC1-E1-F1	GCCTAGTCAAGTCGAGTCTCG	21	57	56.3	193	59
	fNPC1-E2-F	GGCCCCGATGAAACTGAG	18	60	58.4		
	fNPC1-E2-R	GTTCCCAGCGCCCAAGATAAC	21	57	63.2		
3	fNPC1-E3-F	GAGTAATGTGTGTCCTGATTTGAC	24	42	62	162	60
	fNPC1-F3-R	AACTGTAGAGGCAGTGCAAGG	21	52	61.2		
	fNPC1-E3-F2	CTCTGTCCAGGATTCTTCTTTG	22	45	60.1	92	59
	fNPC1-E3-R2	TGTAGAGGCAGCTGCAGGT	19	58	59.5		
	fNPC1-E3-F3	GAGTAATGTGTGTCCTGATTTG	22	41	58.4	175	55
	fNPC1-E3-R3	ACTGTAGAGGCAGTGCA	17	53	52.4		
4	fNPC1-E4-F1	GCTGGCCCTATTATCTGTGA	20	50	58.4	165	58
	fNPC1-E4-R1	GTAACAGGATCAACATAATCTTCAG	25	36	60.9		
	fNPC1-E4-F2	GGCAAAGTCAGTTTCTGAATG	21	43	57.5	166	57
	fNPC1-E4-R2	TAAAAGCTTATTCACCCTCACC	22	41	58.4		
5	fNPC1-E5-F	TTGCCTTCTTCCTCTGCCAG	20	55	60	133	60
	fNPC1-E5-R1	GTACTCAATCCAGTTAGTGGCA	22	55	60		
	fNPC1-E5-F1	ATGACAAAGCCCTGGGACTG	20	55	60	150	58
	fNPC1-F5-R2	AATACACCAAACTTGGGAGTC	21	43	57.5		
6	fNPC1-E6-F	ATTTACTCAACCCCATCCTCCC	22	50	62	185	59
	fNPC1-E6-R	TGCTCACCTGTAGCACCAC	19	58	59.5		
	fNPC1-E6-F1	TCAATTGTTTGTGGCCCAAAGC	22	45	60	183	60
	fNPC1-E6-R1	CAAGATTCTCCAGGGCACAG	20	55	60		
	fNPC1-E6-F2	TGTCAGTGTGCTTAGCACTAG	21	48	59.5	282	57
	fNPC1-E6-R2	CTTGCCCTGGGGAATAAAC	19	53	57.5		
7	fNPC1-E7-F	GGTCTATGGGACTATCAGAGGAAC	24	50	65	168	60

	fNPC1-E7-R	CAAAAGTGCCTACCTCTGTCATTG	24	46	60		
	fNPC1-E8-F1	GACTTTCAGGAATGGCTTGGC	21	50	61	152	59
	fNPC1-E8-R1	GTTTCGGACGCAGAAAGAGC	20	55	60		
	fNPC1-E8-F2	GCATTTGAGGGCTGTCTGAG	20	55	60	124	60
	fNPC1-E8-R2	CAAACACCAGGCCTGAAGAAC	21	52	61		
	fNPC1-E8-F3	GTGTTTGTGCGGGTCACGA	19	58	60	131	59
8	fNPC1-E8-R3	GCCTGGATGATGAGCTGCT	19	58	60		
	fNPC1-E8-F4	GCACTTTGGGCCTTTCTTCC	20	55	60	144	59
	fNPC1-E8-R4	ACGTTACCTGGTGCAAGATC	20	50	58		
	fNPC1-E8-F5	TTCTTCTCCCTGGCCTTCATTG	22	50	62.1	173	60
	fNPC1-E8-R5	GGCTTCCCAGAACGGATTTC	20	55	60.5		
	fNPC1-E8-F6	GTAGCTAACGCTTTCCCTGT	20	50	58.4		
	fNPC1-E9-F1	ATCCAATGTGTTTCTGGGTTTG	22	41	58	131	58
	fNPC1-E9-R1	GGGCCACGCAGATGTCTTG	19	63	62		
9	fNPC1-E9-F2	CTGCCATCGAAAATATCACTGC	22	45	60	142	60
	fNPC1-E9-R2	AGCATGGAATGGCTGTTCTG	22	45	60		
	fNPC1-E9-F3	CTGCACCATTCTGAGCGTG	19	58	60	157	58
	fNPC1-E9-R3	CTGGTGGGCCGAATGATG	18	61	58		
	fNPC1-E10-F	GCTTGTTGTAACTGGGAGTTG	21	48	60	99	59
10	fNPC1-E10-R	CACCTGTTAGTGACAGAACCA	21	48	60		
	fNPC1-E10-F1	CGATACCAGTTTGCTCCATG	20	50	58.4	149	58
	fNPC1-E10-R1	ACCGAATGTACCCAGGCAAG	20	55	60.5		
	fNPC1-E11-F1	CAATAATGCCACAGCCCTTG	20	50	58.4	117	58
11	fNPC1-E11-R	CACACAACAAAGACAGCAACTC	22	45	60		
	fNPC1-E11-F2	CTGCACACTTCTTATCTTCT	20	40	54.3	124	57
	fNPC1-E11-R1	CTGGAGCCTCTCTGTATCAT	20	50	58.4		
	fNPC1-E12-F1	GTCTCACTTAGCTGTCACTAAG	22	45	58	159	57
12	fNPC1-E12-R1	CACCGTTACTTTCACGATTTAG	22	41	58		
	fNPC1-E12-F2	ACTGAGCGAAGTATTGAAGATG	22	41	58	167	58

	fNPC1-E12-R2	CCAGCACAGACACACACACA	20	55	60		
	fNPC1-E13-F1	GGACAAATGATACGCTGACAG	21	48	59.5	155	58
13	fNPC1-E13-R1	CAATGAGGGTGAGGGGGAT	20	50	59.5		
	fNPC1-E13-F2	TGCTCGTTGGGCATCTTTAG	19	58	59.5	162	58
	fNPC1-E13-R2	TGGTCAGATGCCCAGCTCT	19	58	59.5		
	fNPC1-E14-F1	GAAGCAAAGAGATGGCATCG	20	50	58.4	131	57
	fNPC1-E14-R1	CACTTCTCCTAGGACCCT	18	56	56.3		
14	fNPC1-E14-F2	AACTCTGGATCAGCAGCTG	19	53	57.5	137	56
	fNPC1-E14-R2	CCAGGCTTAGAAAACTGTAGG	21	48	59.5		
	fNPC1-E14-F3	CGAGATGAACGTCTTCATGG	20	50	58.4		
	fNPC1-E15-F1	CAGAGTCCTGTGCTGCAG	18	61	58.4	127	58
15	fNPC1-E15-R1	AGAAGGAAGTCGATGAGGAC	20	55	58.4		
	fNPC1-E15-F2	CTCTCTGTTTGCCGGGAT	18	56	56.3	130	56
	fNPC1-E15-R2	GACTCTAATTAGGAGACTGTG	21	43	57.5		
	fNPC1-E16-F	GTCTAGAATCTGACTGAAGCAG	22	45	60.1	144	59
16	fNPC1-E16-R1	CCGAAACAAGCAGCTCTCTG	22	45	60.5		
	fNPC1-E16-F1	GAAGATGGAACCAGTGTCCAG	21	54.4	61.2	155	60
	fNPC1-E16-R	CTTAGAATGCATGGGACAGATC	22	45	60.1		
	fNPC1-E17-F	GCAATCACTGTCTTATAACTCAC	23	39	59.2	115	58
17	fNPC1-E17-R1	GAAAGAGACTGATCCAATCCG	21	48	59.5		
	fNPC1-E17-F1	GTCATTCAGTGTTGCAGTCC	20	50	58.4	114	58
	fNPC1-E17-R	TTTGTTCACACCACTGGCTCA	21	48	59.5		
	fNPC1-E18-F1	CGCCTTGCTTAGTTACTATCAG	22	45	61.1	187	58
	fNPC1-E18-R1	CCGCACACCATGTTCTGC	18	45	60.1		
	fNPC1-E18-F2	TTTGTCCTGGAGGAAGGGCA	20	55	60.5	157	60
18	fNPC1-E18-R2	GCTTCTGAAGTACAAGACAAGG	22	45	60.1		
	fNPC1-E18-F3	GTTCACACTCTCTCCTATTCTT	22	41	60.5	103	59
	fNPC1-E18-R3	CCTTTCAGAGAGGTGTAGTC	20	50	58.4		
	fNPC1-E18-F4	ACTCCTACGTGATGGATTATTTC	23	39	59.2		

19	fNPC1-E19-F	AGACTTCCTGCCTGTGGAG	19	58	59.5	130	58
	fNPC1-E19-R1	CTACAGCAAGAAGACTGAGG	20	50	58.4		
	fNPC1-E19-F1	CGACGATTACTTTGATTGGGTC	22	45	60.1	133	59
	fNPC1-E19-R	CAAAAGTCTTCAGAGAAAAGGG	22	41	58.4		
20	fNPC1-E20-F	CTGGAGACTAATCTTCTGACC	21	48	58.3	143	58
	fNPC1-E20-R1	GCAAACAGAGGCCTCAAGGT	21	48	59.5		
	fNPC1-E20-F1	GCAAACAGAGGCCTCAAGGT	20	55	60.5	123	59
	fNPC1-E20-R	GAGGCAGGCACGTATCTGA	19	58	59.5		
21	fNPC1-E21-F1	TCTGCAGATCCTGTTCTGAC	20	50	58.4	163	58
	fNPC1-E21-R1	CTCATGGCGTCAGTAAAGTC	20	50	58.4		
	fNPC1-E21-F2	CACACCGTGCTTCAGACGT	19	58	59.5	171	58
	fNPC1-E21-R2	GGGTGTGGGCTCTAACCTA	19	58	59.5		
22	fNPC1-E22-F1	GCGAGAATGAGCTGTGACAG	20	55	60.5	178	59
	fNPC1-E22-R1	CATGATCACTGCAGACCACA	20	50	58.4		
	fNPC1-E22-F2	CGTGATTCTCCTGGGCTGT	20	50	59.5	177	58
	fNPC1-E22-R2	GGACACAGGAGTTATGCACTG	21	52	61.2		
23	fNPC1-E23-F	AAGGATGTAACGGAAGCACTT	21	43	57.5	188	56
	fNPC1-E23-R	ACGAGCTGGGCACTTTATG	19	53	57.5		
24	fNPC1-E24-F1	GAGTAGGTTGGTAAGTGCTTTC	22	45	60.1	164	57
	fNPC1-E24-R1	TGGGTGGCTCCCAGTAAGA	19	58	59.5		
	fNPC1-E24-F2	CTTCAGGATGTATTTGGCTATG	22	41	58.4	141	58
	fNPC1-E24-R2	GATCTTTCTTCTCCTTTAGTAGC	23	39	59.2		
25	fNPC1-E25-F	GAACTACCCTGTCGCCTTC	19	58	59.5	180	58
	fNPC1-E25-R	TTCAGAGTCCCCCGACCT	18	61	58.4		

T_m: Melting temperature (salt adjusted), T_a: Annealing temperature used.

Table S2. Primers used to amplify exons and splicing regions of the feline *NPC2* gene.

Exon no.	Name	Primer sequence (5′→3′)	Length (mer)	GC (%)	T_m (ºC)	Amplicon (bp)	T_a (ºC)
1	fNPC2-E1-F	AGGCGGTCGCTTCTTCCT	18	61	58.4	141	58
	fNPC2-E1_R	GCTCCGGCTCACCGCA	16	75	58.4		
2	fNPC2-E1-F2	TCTTGTGACTGAGGCGGTC	19	58	59.5		
	fNPC2-E2-F	CAGGTGGTTTATCTTGCCTC	20	50	58.4	192	58
	fNPC2-E2-R	TGAATTTGAAGTCAAGAGCCAC	22	41	58.4		
3	fNPC2-E3a-F	GCTGGCCCTGTCACTATC	18	61	58.4	137	58
	fNPC2-E3a-R	CACTCTTACAACCATCAGCC	20	50	58.4		
	fNPC2-E3a-F2	CTGTCACTATCTCATTCTCTTTC	23	39	59.2		
	fNPC2-E3b-F	GATGGGCGTAGCAGTTCC	18	61	58.4	170	59
	fNPC2-E3b-R	TCACAAGGCTACCAGTGTC	19	53	57.5		
	fNPC2-E3b-F2	GGCATCCTGATGGGCGT	17	61	57.3	133	56
	fNPC2-E3b-R2	ACCAGTGTCCTGAACAACG	19	53	57.5		
4	fNPC2-E3b-F3	GCTTTGGTGTATGGCATCCT	20	50	58.4		
	fNPC2-E4-F	ACCGGTTCTGGATTGAGGT	19	53	57.5	158	58
	fNPC2-E4-R	AGCACCTCTAGCCTTCAATC	20	50	58.4		
	fNPC2-E4-F1	GTCTGAGGAGTTAGGCAGTT	20	50	58.4		

T_m: Melting temperature (salt adjusted), T_a: Annealing temperature used

Table S3. Primers used to amplify exons and splicing regions of the feline *SMPD1* gene.

Exon no.	Name	Primer sequence (5′→3′)	Length (mer)	GC (%)	T_m (ºC)	Amplicon (bp)	T_a (ºC)
2	fSMPD1-E2-F	GAGCACACCTGTCAATGGCT	20	55	60.5	148	58
	fSMPD1-E2-R	GGTTTCCATGGACCTCAACTA	21	48	59.5		

T_m: Melting temperature (salt adjusted), T_a: Annealing temperature used.
